# Refining Immunogenicity through Intradermal Delivery of Outer Membrane Vesicles against *Shigella flexneri* in Mice

**DOI:** 10.3390/ijms242316910

**Published:** 2023-11-29

**Authors:** Yadira Pastor, Alba Calvo, Josune Salvador-Erro, Carlos Gamazo

**Affiliations:** Department of Microbiology and Parasitology, Navarra Medical Research Institute (IdiSNA), University of Navarra, 31008 Pamplona, Spain; ypastor@unav.es (Y.P.); acalvoba@unav.es (A.C.); jsalvador.1@alumni.unav.es (J.S.-E.)

**Keywords:** *Shigella*, vaccine, outer membrane vesicles, intradermal

## Abstract

Shigellosis remains a global health concern, especially in low- and middle-income countries. Despite improvements in sanitation, the absence of a licensed vaccine for human use has prompted global health organizations to support the development of a safe and effective multivalent vaccine that is cost-effective and accessible for limited-resource regions. Outer Membrane Vesicles (OMVs) have emerged in recent years as an alternative to live attenuated or whole-inactivated vaccines due to their immunogenicity and self-adjuvating properties. Previous works have demonstrated the safety and protective capacity of OMVs against *Shigella flexneri* infection in mouse models when administered through mucosal or intradermal routes. However, some immunological properties, such as the cellular response or cross-protection among different *Shigella* strains, remained unexplored. In this study, we demonstrate that intradermal immunization of OMVs with needle-free devices recruits a high number of immune cells in the dermis, leading to a robust cellular response marked by antigen-specific cytokine release and activation of effector CD4 T cells. Additionally, functional antibodies are generated, neutralizing various *Shigella* serotypes, suggesting cross-protective capacity. These findings highlight the potential of OMVs as a promising vaccine platform against shigellosis and support intradermal administration as a simple and painless vaccination strategy to address this health challenge.

## 1. Introduction

Shigellosis or bacillary dysentery is an enteric foodborne disease caused by the facultative intracellular *Shigella* pathogen. *Shigella* can easily spread from person to person through the fecal-oral route via contaminated water or food, particularly affecting children under five years old in low- and middle-income countries [1]. Despite the sanitation improvements in recent years, epidemiological reports still estimate 270 million shigellosis episodes occur annually, causing over 200,000 deaths worldwide and ranking as the second-leading cause of diarrheal mortality [2]. In high-income regions, such as Europe or the United States, shigellosis is a communicable disease with nearly 450,000 infections each year, according to the Centers for Disease Control and Prevention (CDC), mainly attributed to travel-related diarrhea [3,4]. Treatments typically include the use of antibiotics, but the substantial rise of antibiotic-resistant *Shigella* strains underscores the necessity to develop prophylactic means for preventing the infection, listed by the World Health Organization (WHO) as a priority pathogen for intervention [5,6]. 

Despite ongoing efforts, there is currently no licensed vaccine for human use against *Shigella*, evidencing the challenges to obtaining a successful product against this pathogen. Several factors complicate the development of such a vaccine. Firstly, the global distribution of *Shigella* species with multiple serotypes (*S. flexneri*, *S. sonnei*, *S. boydii,* and *S. dysenteriae*) calls for a multivalent cross-protective vaccine able to address the burden of shigellosis effectively [7]. Additionally, following WHO recommendations, the vaccine must be safe for young children, cost-effective, and accessible to low-income countries. 

The immune response to *Shigella* infection is characterized by the presence of systemic IgGs and mucosal sIgA that are associated with protection in cohort studies [8]. While predominantly functioning as an intracellular pathogen, T-cell-mediated immunity also seems to play a critical role in the course of the infection. However, vaccination efforts have primarily emphasized antibody-mediated responses, leaving cellular immunity moderately understudied. Hence, using a vaccine capable of stimulating cellular and antibody-mediated immunity at the site of infection appears to be the rational approach against shigellosis.

While injectable and oral vaccine candidates have been the main focus, there is a growing interest in exploring alternative routes of administration, such as intradermal needle-free delivery (ID), with the aim of enhancing mucosal immune response and obtaining more suitable vaccines [9]. Needle-free devices for ID administration offer not only easy and painless administration but also unique immunological properties [10]. The skin contains a rich skin-associated lymphoid tissue formed by a diverse population of immune cells, including Langerhans cells, lymphocytes, and dendritic cells, which recognizes antigens and presents them in proximal lymph nodes [11]. Our research group has focused on the potential of Outer Membrane Vesicles (OMVs) as subunit vaccine candidates against shigellosis. These vesicles, containing bacterial antigens and immunostimulatory molecules, are suitable for administering through mucosal routes such as oral, intranasal (IN), or ID routes [12]. In previous studies, we optimized the OMV-vaccine product from a *S. flexneri ΔtolR* mutant, named HT-*ΔtolR*, to be safe and cost-effective, demonstrating the protective capacity of the vaccine in vivo and the biodistribution of this self-adjuvanted vaccine through mucosal lymphoid tissues when administered through nasal, oral, or ID routes [13,14,15].

In this work, we present novel immunological data regarding *S. flexneri* OMVs as acellular vaccine candidates after ID administration in BALB/c mice. Our results demonstrate that skin delivery of HT-*ΔtolR* recruits a diverse and substantial population of immune cells to the site of immunization, eliciting robust T cell- and antibody-mediated immune responses both systemically and at the mucosal site. Importantly, this vaccination induced a bactericidal antibody response against *S. flexneri* and provided protection across different *Shigella* serotypes. Overall, these findings underscore the potential of OMVs as a promising vaccine platform against shigellosis and support the ID administration as a viable strategy for achieving effective and accessible vaccines, particularly in resource-limited regions. This research contributes to the ongoing efforts to develop a *Shigella* vaccine, offering novel alternatives in the fight against this significant public health challenge.

## 2. Results

### 2.1. ID Immunization Recruits Immune Cells into Dermis

**Figure 1:**
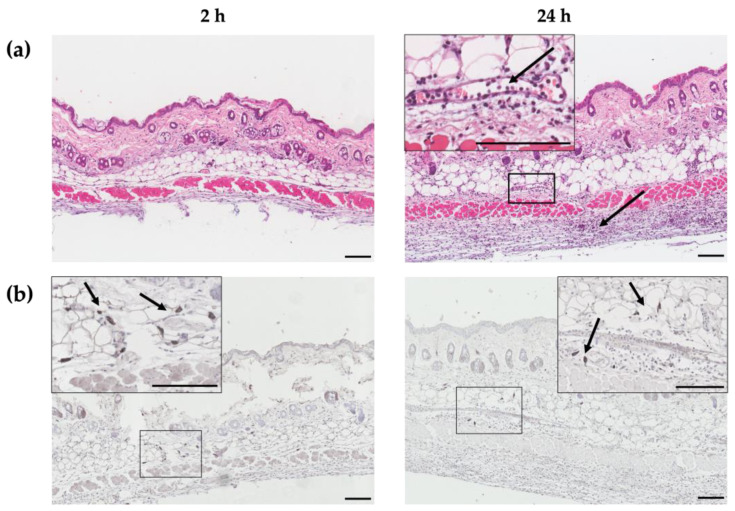
**Histological and immunohistochemical images of dorsal skin sections at 2 and 24 h after HT-*ΔtolR*-intradermal immunization.** Hematoxylin-eosin staining (**a**) and antigen-specific immunohistochemical staining (**b**) of skin sections of a representative female mouse at 2 (**left**) and 24 h (**right**) post-HT-*ΔtolR*-immunization. Specific staining was performed with the Dako EnVision™ antibody complex, followed by incubation with sera from rabbits hyperimmunized with *Shigella flexneri,* and finally incubated with polyclonal goat anti-rabbit IgG. Arrows indicate the recruitment of immune cells, while boxes represent the sections from which the magnifications are displayed. The presence of HT-*ΔtolR* antigens (dark gray) is shown in detail under magnification. Scale bar: 100 µm. (ID: intradermal).

HT-*ΔtolR* antigen delivery into the dermis and the recruitment of leukocytes were demonstrated through histological and immunohistochemical (IHQ) techniques. Skin samples from the site of administration were collected and analyzed at 2 and 24 h post-immunization. A marked infiltration of immune cells was found in the dermis and hypodermis (black arrows, Figure 1a). Antigen-specific staining was detected 2 h post-immunization, decreasing at 24 h (Figure 1b).

### 2.2. ID Administered HT-ΔtolR Vaccine Induces Specific B Cell Response in Mice

**Figure 2:**
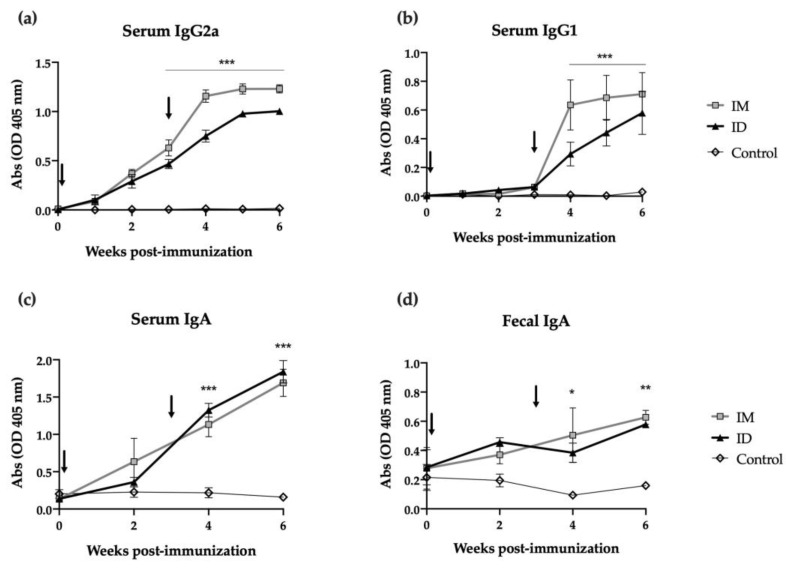
**Antibody immune response induced after skin vaccination of mice with HT-*ΔtolR*.** Specific serum IgG2a (**a**), IgG1 (**b**), IgA (**c**), and mucosal IgA in feces (**d**) levels against HT-*ΔtolR* in immunized BALB/c mice (20 μg). Blood and fecal samples were taken from week 0 to week 6 post-immunization. PBS-immunized mice were used as controls. Sera from immunized mice were diluted 1:100 in PBS-1% BSA, while feces were diluted 1:10 in PBS-T. Arrows indicate immunization time points. Error bars represent SD (n = 6). (* *p* < 0.05, ** *p* < 0.01, *** *p* < 0.001). *(ID: intradermal; IM: intramuscular)*.

Mice were immunized with two ID or intramuscular (IM) doses of HT-*ΔtolR* antigenic complex (20 μg) at day 0 and day 21, and antibody-mediated immune responses were evaluated using ELISA. While antigen specific IgG2a levels increased in both groups from the first immunization (time 0), a second immunization was required in order to elicit specific IgG1 levels (Figure 2a and Figure 2b, respectively). The ID route demonstrated no significant differences when compared to the IM immunization and maintained similar antibody levels even 6 weeks after immunization. Regarding mucosal immune response, strikingly, ID immunized mice showed a significant increase in specific serum IgA (*p* < 0.001) and fecal IgA (*p* < 0.01) after one or two shots of the vaccine. In both cases, this elevated IgA response persisted for two weeks post-boost (Figure 2c,d). 

### 2.3. ID Delivery of HT-ΔtolR Induces Neutralizing Antibodies against Shigella

**Figure 3:**
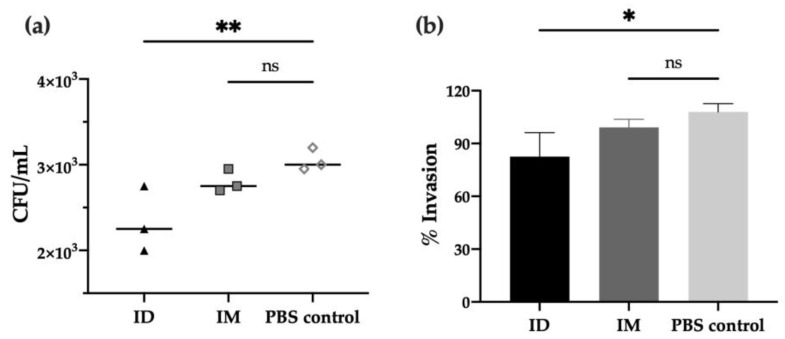
**Invasion capacity of *Shigella flexneri 2a* after serum incubation.** *S. flexneri* 2a bacterial invasion of HeLa cells after treatment with serum of mice immunized with HT-*ΔtolR* either ID or IM, represented as invaded CFU/mL upon gentamicin treatment (**a**) or percentage (%) of invasion referred to serum-free control as 100% invasion (**b**). Heat-inactivated sera at day 42 was used at 1:50 dilution and incubated with bacteria prior to HeLa infection. Error bars represent SD (n = 3). (* *p* < 0.05, ** *p* < 0.01). (*ID: intradermal; IM: intramuscular; ns: no significant*).

In order to characterize the functional activity of antibodies after vaccination, in vitro experiments were performed to evaluate the neutralizing capacity of immunized mice’s sera against different *Shigella* strains. First, the adhesion/invasion assay (AIA), which determines the ability of bacteria to infect HeLa cells after incubation with serum, revealed that antibodies from day 42 post-ID immunization prevented *S. flexneri* invasion compared to PBS control (*p* < 0.01), leading to a 20% reduction of bacterial invasion (*p* < 0.05). Moreover, a slight reduction was observed when compared to the IM group (no significant difference, Figure 3a,b).

**Figure 4:**
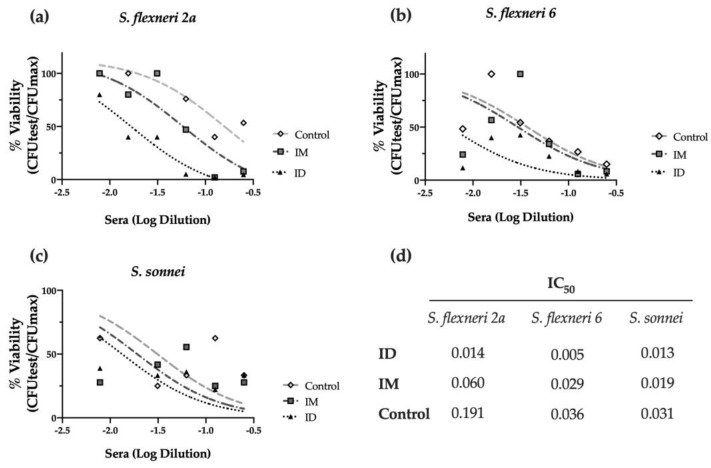
**Serum bactericidal assay against prevalent *Shigella* serotypes.** Complement-mediated bactericidal activity of HT-*ΔtolR* immunized mouse sera against *S. flexneri 2a* (**a**), *S. flexneri* 6 (**b**), and *S. sonnei* (**c**). Results are represented as the percentage of bacterial viability (%) against serially diluted sera from vaccinated mice. Data were normalized as viable CFU/mL with respect to serum-free samples as 100% viability and represented against sera Log dilution. Serum dilutions were able to kill 50% of bacteria in the assay (IC_50_) for control, IM, or ID vaccinated groups are reported (**d**). *p* values were calculated to examine the differences in IC_50_ between the three groups. Experiments were performed three times. (IC_50_: inhibitory concentration 50; ID: intradermal; IM: intramuscular).

Furthermore, we investigated the complement-mediated lysis using mouse sera with the serum bactericidal assay (SBA). As shown in Figure 4a, immunized mouse sera elicited bactericidal activity against *S. flexneri* 2a, decreasing the viability of bacteria (% CFU) when compared to the PBS-vaccinated control group. Strikingly, the ID route elicited the highest serum activity, with an IC_50_ four times lower than the obtained after IM immunization and thirteen times lower than the PBS control group (*p* < 0.001) (Figure 4a,d). This serum also showed bactericidal activity against other relevant *Shigella* serotypes, such as *S. flexneri* 6 (*p* < 0.05), confirming the cross-protective capacity of the HT-*ΔtolR* ID vaccine elicited antibodies (Figure 4b,d). Although the same tendency was observed with *S. sonnei*, the difference was not significant (Figure 4c,d). 

### 2.4. HT-ΔtolR ID Vaccination Elicits Specific T Cell Response in Mice

**Figure 5:**
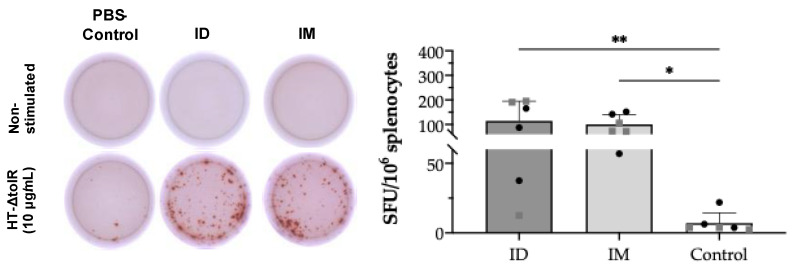
**IFN-γ ELISpot from splenocytes after ID vaccination of BALB/c mice.** Mice were ID or IM immunized with two doses of HT-*ΔtolR* (20 μg). IFN-γ T cells’ response to the HT-*ΔtolR* was assessed using ELISpot from in vitro stimulated splenocytes six weeks post-immunization. After background subtraction, the total number of spots is reported per million splenocytes and compared to the PBS-immunized control group (* *p* < 0.05, ** *p* < 0.01). Males (grey squares) and females (black circles) are represented in each group. Error bars represent SD (n = 6). (SFU: Spot forming units; ID: intradermal; IM: intramuscular).

In order to demonstrate specific T cell responses after skin vaccination, an IFN-γ ELISpot assay was first conducted on splenocytes collected at week 6 post-immunization. After in vitro cell stimulation with HT-*ΔtolR* vesicles (10 μg/mL), the ID immunized group showed significant levels of antigen-specific IFN-γ-producing cells (*p* < 0.01) as compared to the PBS-immunized control group, reaching similar values obtained in the IM control group (*p* < 0.05) (Figure 5). No differences were observed between sexes in any of the analyzed markers.

**Figure 6:**
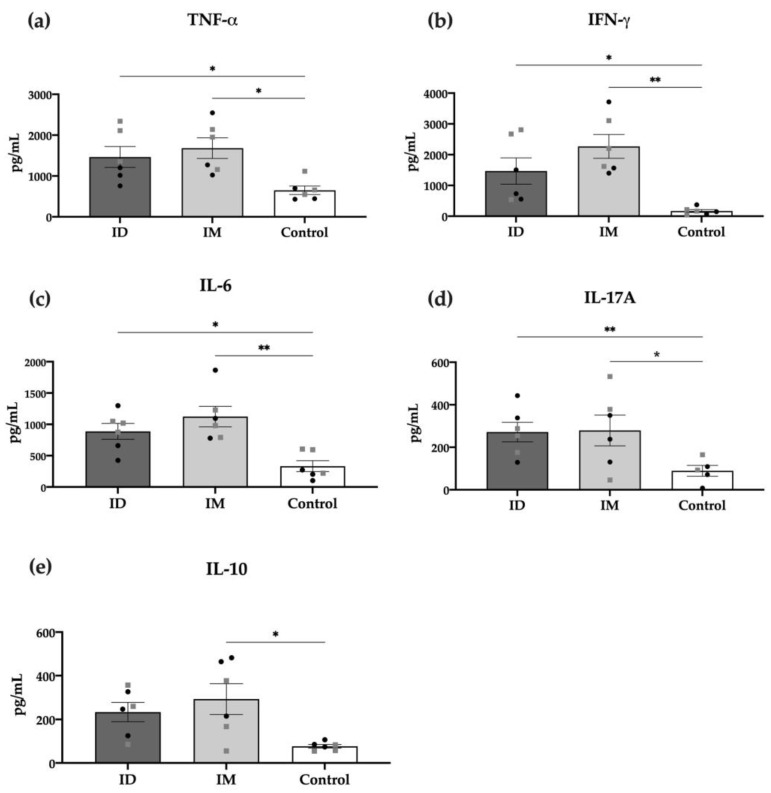
**Cytokine production of splenocytes from immunized mice.** TNF-α (**a**), IFN-γ (**b**), IL-6 (**c**), IL-17 (**d**), and IL-10 (**e**), detected through the cytometric bead array (CBA) in the supernatants of cells re-stimulated for 48 h with 10 μg/mL of HT-*ΔtolR*. Each group represents males (grey squares) and females (black circles). Results are expressed in pg/mL and compared to the PBS-control group (* *p* < 0.05, ** *p* < 0.01). Error bars represent SEM (n = 6). (ID: intradermal; IM: intramuscular; IL: interleukin; TNF: tumor necrosis factor; IFN: interferon).

The specific cellular response was also measured through detecting cytokines in the supernatant of in vitro stimulated splenocytes of vaccinated mice with HT-*ΔtolR* vesicles. As shown in Figure 6, most pro-inflammatory cytokines, such as TNF-α, IFN-γ, IL-6, and IL-17A, were significantly increased in the ID vaccinated group (*p* < 0.05). Interestingly, only the IM but not ID group showed significant levels of the anti-inflammatory cytokine IL-10. No differences were observed between males and females in any of the analyzed cytokines.

**Figure 7:**
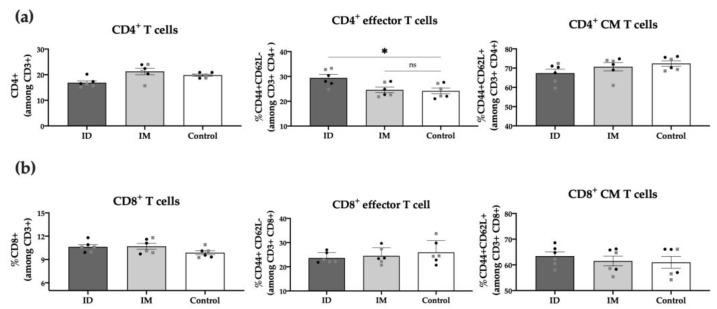
**Spleen T cell populations post-immunization.** Percentage (%) of CD4^+^ T cells (**a**) and CD8^+^ T cells (**b**) among alive CD3^+^ cells in ID and IM HT-*ΔtolR* vaccinated groups determined through flow cytometry. The PBS-control group was used as a negative control. Statistical analysis between the three groups in each panel (ID, IM, and Control) was performed using ordinary one-way ANOVA followed by Dunnett’s multiple comparisons test (* *p* < 0.05). Male (grey squares) and female (black circles) are represented in each group. Error bars represent SEM (n = 6). (ID: intradermal; IM: intramuscular; CM: central memory; ns: no significance).

To further characterize post-vaccination cellular responses, we conducted a phenotypic analysis of the spleen T cell populations 6 weeks post-immunization. Results showed no differences between the ID vaccinated and control groups regarding total CD3^+^, CD4^+^, or CD8^+^ T cells. However, the percentage of CD4^+^ effector T cells (CD44^+^ CD62L^−^) in the ID vaccinated group was significantly higher (*p* < 0.05) compared to unvaccinated control mice, highlighting the role of these cells in response to the HT-*ΔtolR Shigella* vaccine (Figure 7). No differences were observed between males and females in any of the analyzed T-cell markers. 

## 3. Discussion

Ongoing clinical trials for shigellosis vaccines encompass a variety of approaches, such as killed cells and live-attenuated candidates. However, their limited cross-protection among strains, challenging storage requirements, and potential needle-related contamination risks currently impede their widespread use in developing countries [16]. In recent years, ID administration has emerged as a promising alternative due to its easy accessibility and ability to induce both antibody and cellular immune responses. Several studies have compared ID and IM routes, revealing that skin delivery can enhance the immune response, even with lower doses compared to injectable formulations [17]. Although some promising results have been achieved regarding shigellosis, limited information is still available for ID vaccination, particularly using needle-free devices. 

Previous investigations have demonstrated that ID delivery of different *Shigella* virulence factors, such as IpaB, IpaD, SigA, Pic, or Sap, can recruit immune cells into the dermis, inducing both mucosal and systemic immunity [18,19]. However, these proteins required the use of adjuvants to confer protection in mouse models. To address immunogenicity challenges, OMVs have appeared as promising subunit vaccine candidates since they contain multiple bacterial virulence factors and can be modified to overexpress antigens of interest, thereby increasing antigenicity [20]. There are few studies that evaluate the ID administration of OMVs against *Shigella*. Currently, only the *S. sonnei* Generalized Modules for Membrane Antigens (GMMA, GSK Vaccine Institute) have been evaluated for safety and immunogenicity in a Phase 1 clinical trial (clinicaltrial.gov NCT02034500). The vaccine was well-tolerated in healthy adults but resulted in poor immunogenicity, highlighting the need to optimize the dosage and explore needle-free delivery systems deeply [21]. 

In previous studies, we optimized an OMV-vaccine product obtained from the mutant *S. flexneri* 2a *ΔtolR*, named HT-*ΔtolR*, that overexpresses important virulence and immunogenic factors [13,22]. Results confirmed safety and immunogenicity in preclinical models and showed promising protective capacity when administered through nasal [22] or dermal routes [15]. Based on this data, we aimed to complete the immunological profile of the *S. flexneri* OMV-based candidate after needle-free ID vaccination, evaluating the recruitment and activation of innate immune cells and subsequent adaptive immune responses, including both antibody and cell-mediated immunity. 

First, we confirmed through histological studies the delivery of the HT-*ΔtolR* vesicles into the dermis and hypodermis, detecting no signs of irritation or inflammation and confirming the infiltration and presence of a high number of immune cells into the skin layers. We also observed the uptake of the antigenic vesicles by immune cells, corroborating our previous biodistribution results after ID immunization [15]. 

Regarding antibody response, the ID HT-*ΔtolR* immunization elicited high titers of systemic IgG and mucosal IgA, similar to the levels obtained through the IM route. Although we previously demonstrated that the HT-*ΔtolR* vaccine could elicit high specific IgG and IgA levels [13,22], experts agree that antibody-mediated responses need to be more in-depth characterized. To evaluate their functionality, in vitro assays, such as SBA or AIA, are key to better understanding the features of the elicited response regarding its potential correlation with protection [23]. Thus, we demonstrate here that the elicited serotype-specific antibodies were able to neutralize *S. flexneri* 2a successfully, avoiding the invasion of epithelial cells and preventing infection. Moreover, we confirm the complement-dependent bactericidal capacity of the elicited serum antibodies through the SBA assay, which has been considered critical to predicting vaccine efficacy due to its strong association with the reduction in disease post-challenge [24]. Both assays, AIA and SBA, highlight a superior quality in the immune response of the ID route compared to IM vaccination. As previously mentioned, in line with WHO recommendations, the development of a multivalent *Shigella* vaccine is crucial to protect against the most prevalent *Shigella* serotypes, including *S. flexneri* 2a, 3a, 6 and *S. sonnei* [25]. Several works with *Shigella* OMV-vaccine candidates have demonstrated cross-protection against these serogroups in mice when orally or intraperitoneally administered [26,27]. Here, we show for the first time that sera obtained from our OMV complex exhibit cross-neutralizing capacity against different *Shigella* serotypes, and while this finding has to be further confirmed in animal models, it is a promising correlate of protection.

Once the antibody-mediated immune response was confirmed, we intended to evaluate the T-cell response after vaccination. Notably, the function of T lymphocytes is disrupted during a natural infection, modulating the CD4 T cell immune response and migration in vivo [28]. For this reason, a vaccine capable of stimulating the CD4 T cell population may be an interesting way to treat the disease. Our research reveals an increase in effector CD4 T cells (CD44^+^, CD62L^−^), which play a major role in protection. These results align with the significant levels of pro-inflammatory cytokines observed in CBA in both ID and IM groups, suggesting a Th1 response. Among these cytokines, TNF-α, IFN-γ, and IL-6 were detected, which have been identified to be protective during *Shigella* infection [29,30,31]. 

Of note, this study also identified a significant increase in IL-17A in the ID group. The Th17 response has emerged as a key component in the protection against pathogens in mucosal tissues, including *Shigella* [32]. This cytokine induces the release of secretory IgA (sIgA) into the lumen, which was also confirmed in our research. Different groups have reported an increase in IL-17A following vaccination in both mice [33] and humans [29], underscoring the significance of Th1/Th17 cytokine responses as an important indicator of vaccine protective capacity.

Overall, this study contributes novel immunological insights into OMVs as potential vaccine candidates against shigellosis and highlights their promising protective capacity when administered through the ID route using needle-free devices. This strategy provides a safe, convenient, painless, and accessible delivery system that could be highly affordable for low-income countries.

## 4. Materials and Methods

### 4.1. Animal Ethics Statement

Mice used in this study were treated in accordance with institutional guidelines for the treatment of animals (Ethical Committee for Animal Experimentation of the University of Navarra, Spain; Protocol ref. CEEA 027/20).

### 4.2. Preparation of Outer Membrane Vesicles

As previously described, an antigenic complex based on OMV was obtained from the *S. flexneri ΔtolR* mutant strain [13]. The parental strain *S. flexneri* 2a is a clinical isolate from the “Clínica Universidad de Navarra”, Spain. Both the wildtype and the mutant strain were cultured on tryptone soy agar (TSA, Biomerieux, Madrid, Spain) or in tryptone soy broth (TSB, Biomerieux). Incubations were performed at 37 °C with shaking (140 rpm) to log phase (OD 600 0.3). Bacterial cultures were heat-inactivated (HT) via flowing steam (100 °C) for 15 min. After centrifugation at 6000× *g* for 20 min, the HT-*ΔtolR* containing supernatants were filtered (0.22 μm) and purified using 100 kDa-tangential filtration (Millipore, Damstadt, Germany). The retenant was then harvested and centrifugated (51,000× *g*, 1 h), and pellets were resuspended in deionized water, lyophilized, and stored at room temperature (RT) until use.

### 4.3. Mice Immunization

Nine-week-old male and female BALB/c mice (20 ± 1 g) were separated into two randomized groups of 6 animals and immunized with either ID or IM with 20 μg of HT-*ΔtolR* extracts at days 0 and 21. For ID administration, 50 μL of inoculum was delivered into the upper right thigh of the back (shaved the day before) using the MicronJetTM™ microneedle system (NanoPass Technologies, Ness Ziona, Israel). The microneedle was inserted at 45°, and the inoculum was applied slowly and observed until the bleb was totally absorbed. A group of ID-immunized mice with PBS was included as a negative control. For IM immunization, the same volume of inoculum was used. Blood and stools were taken before immunization (time 0) and once or twice per week, respectively, up to 6 weeks post-immunization. At the end of the experiment, spleens were collected for assessing B and T cell responses. No animals were excluded from the study. 

### 4.4. Histology and Immunohistochemistry

To analyze the vaccine delivery into the dermis and recruitment of immune cells after ID immunization (see above), mice were sacrificed at 2 or 24 h (n = 3/group). The targeted skin portion was cut off and fixed in 4% formaldehyde (Panreac, Madrid, Spain) for 48 h. Tissues were then incubated in ethanol 70% for 72 h, paraffin-embedded, and sectioned into 4 μm-thickness slides. In order to evaluate histological changes, tissue sections were stained with hematoxylin and eosin and observed using light microscopy.

Immunohistochemistry (IHQ) was used to detect and localize the ID of the inoculated vaccine. Specific staining was performed with the Dako EnVision™ antibody complex (Agilent, Santa Clara, CA, USA) following the manufacturer’s instructions. Briefly, slides were dried at 60 °C for 30 min and then hydrated with decreasing concentrations of ethanol. After washing with TRIS buffer (TBS: 0.55 M, TRIS 0.05 M, NaCl 0.5 M, pH 7.4), samples were treated with hydrogen peroxide for 10 min and then blocked for 30 min with normal goat serum. Slides were then incubated with sera from rabbits hyperimmunized with *S. flexneri* [34] for 4 h at RT, washed, and incubated with polyclonal goat anti-rabbit IgG conjugated with peroxidase for 30 min at RT. Glucose oxidase was used for revealing, and hematoxylin was used for contrast staining. Sections were dehydrated and mounted, and slides were imaged using optical microscopy.

### 4.5. Splenic Cells Processing

Spleens were collected and kept in cold PBS, and cells were processed for cellular immune response assessment. Briefly, spleen cells were harvested via mashing the tissue under a genlteMACS^TM^ dissociator (Miltenyi Biotec, Bergisch Gladbach, Alemania) and subsequently passing them through 0.4 μm nylon cell strainers (Falcon^®^, Becton Dickinson, Franklin Lakes, NJ, USA). Red blood cells were then removed using ammonium-chloride-potassium lysing buffer (ACK, Gibco^®^, Damstadt, Germany) for 5 min and then washed twice with PBS, and centrifuged. Cells were resuspended in warm RPMI-1640 medium supplemented with 10% fetal bovine serum (FBS) and 1% penicillin-streptomycin (Gibco^®^) and counted for the following cellular assays. 

### 4.6. Specific Antibody-Mediated Responses

Specific IgG1, IgG2a, and IgA antibodies in sera, as well as specific mucosal IgA in the feces of immunized mice, were determined using an ELISA assay. Briefly, 96-well plates (MaxiSorp; Nunc, Waltham, MA, USA) were coated with HT-*ΔtolR* vesicles (1 μg/mL) in coating buffer (60 mM carbonate buffer, pH 9.6). Unspecific binding sites were blocked with 1% bovine serum albumin (BSA) in PBS for 1 h at RT. Sera from immunized mice were diluted 1:100 in PBS with 1% BSA and incubated for 4 h at RT, while feces were diluted 1:10 in PBS Tween20 (PBS-T). Of note, for detecting IgA in sera, Protein G Dynabeads^®^ (Life Technologies AS, Damstadt, Germany) was used according to the manufacturer’s instructions to remove blocking IgG from the samples prior to the 4 h incubation. After washing with PBS-T, class-specific goat anti-mouse IgG1, IgG2a, or IgA (Sigma-Aldrich, Madrid, Spain) conjugated antibodies were added and incubated for 1 h at RT. Absorbance was measured (O.D. 405 nm) with an ELISA plate reader (Tecan, Grodig, Austria) after incubation with H_2_O_2_-ABTS substrate-chromogen for 15 min at RT.

### 4.7. Adhesion/Invasion Assay

To elucidate the neutralizing capacity of antibodies against *Shigella* strains, an adhesion and invasion assay (AIA) was performed with some modifications [34]. Briefly, HeLa cells were seeded at 1 × 10^5^ cells/well in 24-well plates and incubated ON in RPMI-1640 medium (Gibco^®^) supplemented with 10% FBS and 1% penicillin-streptomycin (Gibco^®^) at 37 °C, 5% CO_2_. Pre-cultures of *Shigella* strains were grown ON in LB medium and diluted to reach 1 × 10^7^ CFU/well [multiplicity of infection (MOI) of 100:1]. Prior to cell infection, serial dilutions of heat-inactivated sera (56 °C, 30 min) were prepared and incubated with bacteria for 30 min at 37 °C under shaking. HeLa cells were then washed twice with PBS and resuspended in antibiotic-free complete media. A volume of 60 μL of serum-bacteria mixture was added to the cells in a final volume of 300 μL, and plates were centrifuged (900× *g*, 10 min) and incubated at 37 °C and 5% CO_2_ for 2 h. Cell cultures were then washed, and fresh medium containing 50 μg/mL gentamicin was added and incubated for 1 h at 37 °C, 5% CO_2_. Finally, cells were lysed with a 1% Triton X-100 solution (Sigma, Madrid, Spain), and the lysates were serially diluted and plated on TSA for viable colony counting. Results were referred to serum-free controls as 100% invasion. 

### 4.8. Serum Bactericidal Assay

The conventional serum bactericidal assay (SBA) [35] was performed to confirm the functional properties of antibodies generated after vaccination. Log-phase bacterial cultures were prepared, as described above, and diluted to 1 × 10^6^ CFU/mL in LB broth. Heat-inactivated (HI) sera (70 μL) were serially diluted in 96-well round bottom sterile plates (Corning^®^) with bacteria (10 μL) and human complement as an external source of complement (20 μL, Sigma-Aldrich) in a final volume of 100 μL. Of note, prior to the assay, the optimal percentage of complement was determined for *S. flexneri* 2a, and thus, we used 20% of human complement, which lacked intrinsic bactericidal activity. The assay also included negative controls, represented by bacterial samples without any antibody source. The plates containing the reaction mixtures were incubated for 3 h at 37 °C under shaking. At time 0 and the end of incubation, serial dilutions of the reactions were plated on TSA and incubated ON for counting. Results were expressed as serum dilution necessary to obtain a 50% reduction of CFU (IC_50_) in relation to the maximum growth of bacteria obtained in the test (serum-free samples). Data were adjusted following a dose-response curve using the least squares regression method provided by GraphPad Prism 9 software to calculate IC_50_ values.

### 4.9. Cellular Immune Responses

The frequency of IFN-γ-releasing specific cells was detected in fresh mice splenocytes using the ELISpot assay (Becton Dickinson) following the manufacturer’s instructions. Briefly, ELISpot plates were coated with a capture anti-mouse IFN-γ antibody and incubated at 4 °C ON. Cells were seeded at 0.8 × 10^6^ cells/well and stimulated with 10 μg/mL of HT-*ΔtolR* vesicles at 37 °C, 5% CO_2_ for 24 h. Plates were then incubated with an anti-IFN-γ detection antibody in PBS-0.5% FBS for 2 h at RT and then washed, and Streptavidin-HRP 1/1000 in PBS-0.5% FBS was added for 1 h. Finally, samples were washed and developed using AEC substrate solution (BD™). Spot-forming cells were counted using an ImmunoSpot automated counter (CTL-Immunospot; Bonn, Germany). Cells stimulated with the mitogens phorbol Myristic Acid (PMA) and ionomycin (Sigma) at 0.1 and 4 μg/mL, respectively, were used as positive controls.

Analysis of cytokines from immunized mice was measured in culture supernatants of splenocytes stimulated for 48 h with 10 μg/mL of HT-*ΔtolR* using the cytometric bead array (CBA) mouse Th1/Th2/Th17 Cytokine kit (Becton Dickinson), following the manufacturer’s instructions and analyzed on an Attune acoustic focusing cytometer (Applied Biosystems, Waltham, MA, USA). Standard curves were determined for each cytokine in a range of 20–5000 pg/mL. The following cytokines were measured: IFN-γ, TNF-α, IL-6, IL-10, and IL-17A.

### 4.10. Flow Cytometry Analysis

In order to analyze immune cell populations in the spleens of mice, 1 × 10^6^ cells per well were incubated with purified anti-mouse CD16/32 (Biolegend^®^) in 50 μL of FACS buffer, San Diego, California, USA (PBS supplemented with 2% FBS and 2.5 mM EDTA) per well for 10 min on ice to block non-specific binding. Afterward, cells were stained with propidium iodide used for assessing cell viability (Invitrogen, Waltham, Massachusetts, USA) and the different fluorochrome-conjugated antibodies: CD3-PE/Cy7 (clone 17A2, Biolegend^®^), CD4-FITC (clone RM4-4, Biolegend^®^) CD44-PerCP/Cy5.5 (clone IM7, Biolegend^®^) and CD62L-APC (clone MEL-14, Becton Dickinson) and analyzed with an Attune acoustic focusing cytometer (Applied Biosystems). Cytometric analysis was performed using the FlowJo V10 software. Analyses of leukocyte populations were conducted according to predefined gate settings (full gating strategy is shown in Figure S1). 

### 4.11. Statistical Analysis

Statistical analysis was carried out using the parametric t-student test, one-way ANOVA, or the non-parametric Mann–Whitney U test, as required, following multiple comparison tests. *p* values < 0.05 were considered statistically significant. All calculations were performed using GraphPad Prism 9^®^ software (San Diego, CA, USA). 

## Data Availability

Data is contained within the article and supplementary materials.

## Supplementary Materials

The following supporting information can be downloaded at: https://www.mdpi.com/article/10.3390/ijms242316910/s1.
