# Supplementary material for: Refining Immunogenicity through Intradermal Delivery of Outer Membrane Vesicles against Shigella flexneri in Mice
Source: Int J Mol Sci. 2023 Nov 29;24(23):16910. doi: 10.3390/ijms242316910 (PMC10706920; doi:10.3390/ijms242316910)
Supplement: Supplementary file 1 [file ijms-24-16910-s001.zip › ijms-2719751-supplementary.pdf]

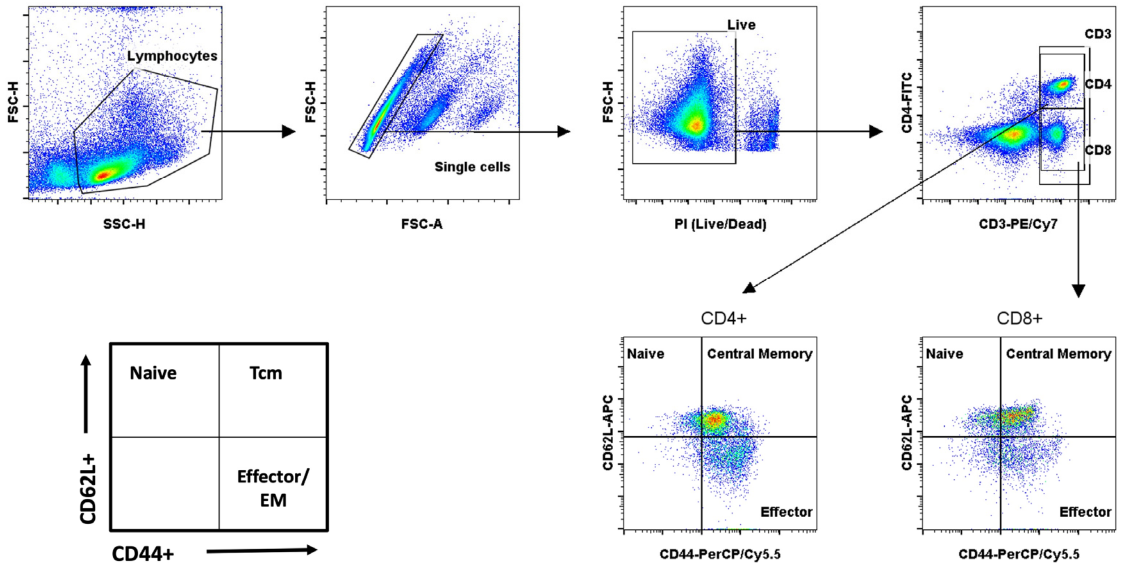

Figure S1. Gating strategy for mice splenocyte populations 6 weeks post-immunization. T-lymphocytes were selected from a forward scatter height vs side scatter-height dot plot, and single cells were subsequently selected in a forward scatter-area vs forward scatter height dot plot. From living cell population, T cells were selected by $CD3^{+}$ expression and $CD8^{+}$ cytotoxic and $CD4^{+}$ helper T cells were identified by a CD8 vs CD4 dot plot. From each population, effector or central memory T cells were identified by a CD44 vs CD62L dot plot. (*Tcm: T central memory cells; EM: effector memory*).
